# Genotoxicity effect of methyl-tertiary butyl ether on rat lymphocytes using comet assay

**Published:** 2020-05-25

**Authors:** Shima Alishahi, Zahra Zendeh-Boodi, Mostafa Saadat

**Affiliations:** 1 Department of Biology, College of Sciences, Shiraz University, Shiraz 71467-13565, Iran

## ⁯⁯

***Dear Editor,***

Methyl-tertiary butyl ether (MTBE), an oxygenated compound, is added to gasoline in order to reduce air pollutants. MTBE alters enzyme activity or mRNA levels of antioxidant enzymes (Elovaara et al., 2007[11]; Khalili et al., 2015[12]; Badr et al., 2016[1]; Badr and Saadat 2016[3], 2018[2]) and oncogenes (Zhou et al., 1999[14]). It has been reported that elevation of cancer incidence is associated with exposure to MTBE (Belpoggi et al., 1995[4]). Therefore, its usage has been limited in several countries. However, it is still used in several Middle East countries. In many countries, people are exposed to very low levels of MTBE by drinking contaminated water (Brown 1997[6]). In a majority of previous reports, researchers have investigated the effects of high or very high doses of MTBE on experimental animals (de Peyster et al., 2014[9]; Dongmei et al., 2009[10]; Khalili et al, 2015[12]; Badr et al., 2016[1]). It should be noted that low levels of MTBE significantly alter the gene expression levels of GSTs (Badr and Saadat 2018[2]).

Although many investigations concerning genotoxicity of MTBE and its metabolites have been carried out, the potential risk of human mutagenicity, is still a matter of debate among toxicologists (Bogen et al., 2015[5]). The purpose of the present experiment is to examine the potential of MTBE at low doses in inducing DNA damage condition using alkaline comet assay.

A total of 24 adult Wistar male rats (180-200 g) were purchased from the animal house of Shiraz University of Medical Sciences (Iran). Animals were housed in plastic cages under standard animal house conditions with a 12 h light/dark cycle and a temperature of 25 ± 2 °C, received standard pellet food, and tap water was available *ad libitum*. After acclimation period (10 days), the animals were randomly divided into four equal experimental groups. Group 1 (control) was treated with distilled water and groups 2, 3 and 4 received 5, 10 and 20 mg/kg/day MTBE in distilled water, respectively. The treatments were done through oral gavage for 30 consecutive days. 

At the end of the exposure period, animals were anesthetized with ether and blood samples were obtained from their heart. Lymphocytes were separated by density centrifugation at 1000×*g *for 30 min at the room temperature over a layer of Ficoll and then the cell suspension was adjusted to 10^4^-10^6^ cells/ml. For positive control, 50 μL of the cell suspension obtained from untreated rat was mixed with 1 μM H_2_O_2_ and incubated for 5 min at 4 °C. 

The comet assay was performed under alkaline conditions as described previously (Olive and Banáth, 2006[13]) with some modifications. Thereafter, images of 50 randomly chosen cells were taken per sample with Nikon fluorescence microscope, and then, indices of cellular DNA damage were measured using TriTek CometScore V 2.0 software. Indices of DNA damage were the tail length (TL), tail DNA percent (TD), and tail moment (TM).

**Figure 1:**
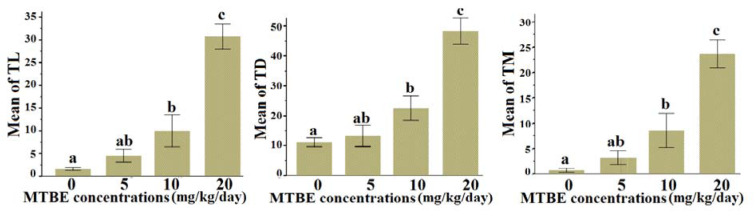
Effects of MTBE on DNA damage indices including the tail length (TL), tail DNA percent (TD), and tail moment (TM). Values are means ± SE. Same alphabet means no significant differences between groups.

Statistical analysis indicated that the TL, TD and TM indices in the positive controls which were treated with 1 μM H_2_O_2_ showed maximum levels of DNA damage, while the control group revealed very low DNA damage. One-way analysis of variance demonstrated that there were significant statistical differences between the experimental groups for the LT (F=31.19; df=3, 20; P<0.001), TD (F=22.22; df=3, 20; P<0.001), and TM (F=20.35; df=3, 20; P<0.001) indices. The post hoc Duncan test revealed that the means of the study indices were significantly increased in the groups that received 10 and 20 mg/kg/day MTBE compared with the control levels (Figure 1(Fig. 1)). Pearson correlation analysis revealed that there were significant positive correlations between MTBE concentrations and the TL (r=+0.882, df=22, P<0.001), TD (r=+0.851, df=22, P<0.001) and TM (r=+0.852, df=22, P<0.001) indices. It means that the amount of DNA damage increases as a function of MTBE concentration. 

MTBE has been recently reported to induce chromosomal aberration in bone marrow cells of rats, orally administered with this chemical at high concentration (Darwish and Mosallam 2019[8]). A significant increase in the extent of DNA damage was observed in human lymphocytes treated with MTBE at low doses (Chen et al., 2008[7]). Our present findings indicate that MTBE can induce DNA strand breaks even at low doses under *in vivo* condition. 

Considering that MTBE is metabolized to the known weak carcinogens, tert-butanol and formaldehyde (Belpoggi et al., 1995[4]), and in order to find the real association between low levels of MTBE and possible alterations in incidence rates of human cancers, further research in this field is required.

## Acknowledgement

This work was supported by the Shiraz University (97GCU2M1741), Iran.

## Conflict of interest

None.
